# Supplementary material for: Effect of Serum and Oxygen on the In Vitro Culture of Hanwoo Korean Native Cattle-Derived Skeletal Myogenic Cells Used in Cellular Agriculture
Source: Foods. 2023 Mar 24;12(7):1384. doi: 10.3390/foods12071384 (PMC10093918; doi:10.3390/foods12071384)
Supplement: Supplementary file 1 [file foods-12-01384-s001.zip › supplementary tables.pdf]

Supplementary Table S1. Primer list used for real time PCR.

Genes		Sequence	Accession No.
PAX7	Forward	AGGACGGCGAGAAGAAAGC	XM_019984705.1
	Reverse	CCCTTTGTCGCCCAGGAT	
MYF5	Forward	CAGCCTCTCTCTCCCCAGTTG	NM_174116.1
	Reverse	AGGCCCCTGGAGTTGCA	
MYOD1	Forward	CACGTCTAGCAACCCAAACCA	AB110599.1
	Reverse	ATGGCGTTGCGCAGGAT	
MYOG	Forward	GACGCCATCATCCATGTTCTAC	NM_173881.2
	Reverse	GGGCATCAGCACCGAAGT	
MYF6	Forward	GGCGGCGGCTCAAGA	AB110601.1
	Reverse	AACGAGGCCTTCGAGG	
MYH1	Forward	GCCAGACTGTAGAGCAGGTATATAACG	NM_174117.1
	Reverse	GCAACCATCCACAGGAACATC	
MSTN	Forward	CGGCTCCTTGGAAGACGAT	NM_001001525.3
	Reverse	CTCCGTGGGCATGGTAATG	
TERT	Forward	GCAGGTCCTACATCCAGTGTCA	NM_001046242.1
	Reverse	TCCATGTCCCCATAGCAGAAG	
CDKN1A	Forward	CTCCCAGGGCCGGAAA	NM_001098958.2
	Reverse	GCGTTTGGAGTGGTAGAAATCTG	
TP53	Forward	TTACGCGCGGAGTATTTGG	NM_174201.2
	Reverse	GGCACCACCACACTGTGTCTA	
MYC	Forward	CATCCTGTCGGTCCAAGCA	NM_001046074.2
	Reverse	CTCTTCTGCAACACGTCTATTTCTG	
BAX	Forward	CGCATCGGAGATGAATTGG	NM_173894.1
	Reverse	CCACAGCTGCGATCATCCT	
BCL2	Forward	GGAGCTGTATGGCCCTAGCAT	NM_001166486.1
	Reverse	CCTTCAGAGACAGCCAGGAGAA	
NOX1	Forward	TGATCCTGCCTCCTACTGCAA	NM_001191340.1
	Reverse	GTGCAAGGATCCATTTCCAAGA	
GPX1	Forward	TTTGGGCATCAGGAAAACG	NM_174076.3
	Reverse	CGGACGTACTTCAGGCAATTC	
SOD1	Forward	GTTGGAGACCTGGGCAATGT	NM_174615.2
	Reverse	ACAATATCCACGATGGCAACAC	
SOD2	Forward	GGGTGATGTTACAGCTCAGATAGCT	NM_201527.2
	Reverse	GGCCCCCACCGTTGA	
SOD3	Forward	TGGAGGCCTTCTTCCACCTT	NM_001082610.1
	Reverse	TGGATGGCACGGTTTGTG	
HIF	Forward	GATCTCGTCGAAGTAAAGAGTCTGAA	NM_174339.3
	Reverse	AGCCTTATCAAGATGCGAGCTT	
MAPK-14 (p38)	Forward	TTTTGCACGTCTTGCTATTTGAG	NM_01102174.1
	Reverse	AGGTGACACGGGTTCTGAACTAC	
MAPK-3 (ERK1)	Forward	GCACATTGCGAGAGATTCAGATT	NM_001110018.1
	Reverse	GGTGCCCGCAGAATGTCT	
MAPK-1 (ERK2)	Forward	GTGACCTCAAACCTTCCAACCT	NM_175793.2
	Reverse	GGCCAAGCCAAAGTCACAGA	
MTOR	Forward	CATGCTTCGTGTCTTCATGCA	XM_002694043.6
	Reverse	GGGCGTCAAATAACTTCACGAT	

Supplementary Table S2. Interaction effect of FBS and oxygen concentrations on the proliferation of SMCs by Two-way ANOVA.

	Day	Sum of	df	Mean Square	F	p	η^2	$\eta^2 p$
**2 days**	FBS	0.00173	2	0.00087	12	0.001	0.433	0.667
	Oxygen	0.00109	1	0.00109	15.08	0.002	0.272	0.557
	FBS ✻ Oxygen	3.11E-04	2	0.00016	2.15	0.159	0.078	0.264
	Residuals	8.67E-04	12	0.00007				
**4 days**	FBS	0.0313	2	0.01565	30.62	< .001	0.301	0.836
	Oxygen	0.05894	1	0.05894	115.3	< .001	0.568	0.906
	FBS ✻ Oxygen	0.00748	2	0.00374	7.32	* 0.008	0.072	0.549
	Residuals	0.00613	12	0.00051				
**6 days**	FBS	0.4511	2	0.22557	58.6	< .001	0.336	0.907
	Oxygen	0.5512	1	0.55125	143.2	< .001	0.41	0.923
	FBS ✻ Oxygen	0.2956	2	0.14782	38.4	* < .001	0.22	0.865
	Residuals	0.0462	12	0.00385				
**8 days**	FBS	4.123	2	2.06150	98.1	< .001	0.432	0.942
	Oxygen	4.59	1	4.59050	218.5	< .001	0.481	0.948
	FBS ✻ Oxygen	0.576	2	0.28780	13.7	* < .001	0.06	0.695
	Residuals	0.252	12	0.02100				
**10 days**	FBS	14.68	2	7.33900	51.5	< .001	0.48	0.896
	Oxygen	7.97	1	7.97300	55.9	< .001	0.261	0.823
	FBS ✻ Oxygen	6.19	2	3.09600	21.7	* < .001	0.203	0.783
	Residuals	1.71	12	0.14300				

* $p < 0.05$

Supplementary Table S3. Interaction effect of FBS and oxygen concentration of myogenetic-transcription factor genes in cells cultured in vitro with GM for 7 days by two-way ANOVA.

Genes		Sum of Squares	df	Mean Square	F	p	η^2	$\eta^2 p$
PAX7	FBS	0.377	2	0.18849	98.44	< .001	0.212	0.916
	Oxygen	0.0262	2	0.01308	6.83	0.006	0.015	0.432
	FBS ✻	1.3395	4	0.33488	174.89	< .001	0.754	0.975
	Residuals	0.0345	18	0.00191				
MYF5	FBS	23.1057	2	11.55287	7998.1	< .001	0.935	0.999
	Oxygen	0.1401	2	0.07003	48.5	< .001	0.006	0.843
	FBS ✻	1.4502	4	0.36255	251	< .001	0.059	0.982
	Residuals	0.026	18	0.00144				
MYOD1	FBS	153.9932	2	76.99658	69529	< .001	0.986	1
	Oxygen	0.3456	2	0.1728	156	< .001	0.002	0.945
	FBS ✻	1.7599	4	0.43998	397	< .001	0.011	0.989
	Residuals	0.0199	18	0.00111				
MYOG	FBS	494.5958	2	247.29789	19466	< .001	0.994	1
	Oxygen	0.6007	2	0.30036	236	< .001	0.001	0.963
	FBS ✻	2.1354	4	0.53386	420	< .001	0.004	0.989
	Residuals	0.0229	18	0.00127				
MYF6	FBS	1.5462	2	0.7731	193.09	< .001	0.405	0.955
	Oxygen	0.00447	2	0.00223	0.558	0.582	0.001	0.058
	FBS ✻	2.19213	4	0.54803	136.88	< .001	0.575	0.968
	Residuals	0.07207	18	0.004				
MYH1	FBS	141.922	2	70.9612	1676.9	< .001	0.953	0.995
	Oxygen	5.111	2	2.5556	60.4	< .001	0.034	0.87
	FBS ✻	1.053	4	0.2633	6.22	0.003	0.007	0.58
	Residuals	0.762	18	0.0423				

* $p < 0.05$

Supplementary Table S4. Interaction effect of FBS and oxygen concentration of myogenetic-transcription factor genes in cells cultured in vitro with GM for 14 days by two-way

Genes		Sum of Squares	df	Mean Square	F	p	η^2	η^2p
PAX7	FBS	1.9174	2	0.95868	398.2	< .001	0.271	0.978
	Oxygen	0.0486	2	0.02431	10.1	0.001	0.007	0.529
	FBS ✻ Oxygen	5.0674	4	1.26684	526.2	< .001	0.716	0.992
	Residuals	0.0433	18	0.00241				
MYF5	FBS	11.4128	2	5.70641	2399.89	< .001	0.703	0.996
	Oxygen	0.0443	2	0.02214	9.31	0.002	0.003	0.508
	FBS ✻ Oxygen	4.7439	4	1.18597	498.77	< .001	0.292	0.991
	Residuals	0.0428	18	0.00238				
MYOD1	FBS	2.7015	2	1.35077	793	< .001	0.344	0.989
	Oxygen	0.4608	2	0.2304	135	< .001	0.059	0.938
	FBS ✻ Oxygen	4.6588	4	1.16469	684	< .001	0.593	0.993
	Residuals	0.0307	18	0.0017				
MYOG	FBS	14.6482	2	7.324	8635	< .001	0.832	0.999
	Oxygen	1.1668	2	0.583	688	< .001	0.066	0.987
	FBS ✻ Oxygen	1.771	4	0.443	522	< .001	0.101	0.991
	Residuals	0.0153	18	8.48E-04				
MYF6	FBS	2.8022	2	1.40108	399.5	< .001	0.373	0.978
	Oxygen	0.3702	2	0.18509	52.8	< .001	0.049	0.854
	FBS ✻ Oxygen	4.2728	4	1.0682	304.6	< .001	0.569	0.985
	Residuals	0.0631	18	0.00351				
MYH1	FBS	217.382	2	108.6908	8952.6	< .001	0.971	0.999
	Oxygen	3.555	2	1.7773	146.4	< .001	0.016	0.942
	FBS ✻ Oxygen	2.638	4	0.6595	54.3	< .001	0.012	0.923
	Residuals	0.219	18	0.0121				

* $p < 0.05$

Supplementary Table S5. Interaction effect of FBS and oxygen concentration on proliferation and apoptosis genes in cells cultured in vitro with GM for 14 days.

Genes		Sum of	df	Mean	F	p	η^2	$\eta^2 p$
TERT	FBS	8.2368	2	4.118	6178	< .001	0.817	0.999
	Oxygen	0.6462	2	0.323	485	< .001	0.064	0.982
	FBS ✻	1.1879	4	0.297	445	< .001	0.118	0.99
	Residuals	0.012	18	6.67E-04				
CDKN1A(P21)	FBS	20.39867	2	10.199	21684	< .001	0.786	1
	Oxygen	1.99103	2	0.996	2116	< .001	0.077	0.996
	FBS ✻	3.56404	4	0.891	1894	< .001	0.137	0.998
	Residuals	0.00847	18	4.70E-04				
TP53	FBS	5.84436	2	2.922	8576	< .001	0.773	0.999
	Oxygen	0.32202	2	0.161	473	< .001	0.043	0.981
	FBS ✻	1.39136	4	0.348	1021	< .001	0.184	0.996
	Residuals	0.00613	18	3.41E-04				
MYC	FBS	5.5214	2	2.761	12219	< .001	0.483	0.999
	Oxygen	4.52136	2	2.261	10006	< .001	0.395	0.999
	FBS ✻	1.39484	4	0.349	1543	< .001	0.122	0.997
	Residuals	0.00407	18	2.26E-04				
BAX	FBS	3.17472	2	1.587	7026	< .001	0.643	0.999
	Oxygen	0.62512	2	0.313	1383	< .001	0.127	0.994
	FBS ✻	1.13195	4	0.283	1253	< .001	0.229	0.996
	Residuals	0.00407	18	2.26E-04				
BCL2	FBS	0.03721	2	0.0186	56.4	< .001	0.006	0.862
	Oxygen	2.00501	2	1.0025	3041.3	< .001	0.331	0.997
	FBS ✻	4.01699	4	1.0042	3046.6	< .001	0.662	0.999
	Residuals	0.00593	18	3.30E-04				

* $p < 0.05$

Supplementary Table S6. Interaction effect of FBS and oxygen concentration on reactive oxygen species (ROS) genes in cells cultured in vitro with GM for 14 days.

Genes		Sum of Squares	df	Mean Square	F	p	η^2	$\eta^2 p$
NOX4	FBS	2.4779	2	1.239	7965	< .001	0.411	0.999
	Oxygen	0.52676	2	0.263	1693	< .001	0.087	0.995
	FBS ✻ Oxygen	3.01773	4	0.754	4850	< .001	0.501	0.999
	Residuals	0.0028	18	1.56E-04				
GPX1	FBS	6.8595	2	3.42975	14936	< .001	0.999	0.999
	Oxygen	8.30E-04	2	4.15E-04	1.81	0.193	0	0.167
	FBS ✻ Oxygen	0.00468	4	0.00117	5.1	0.006	0.001	0.531
	Residuals	0.00413	18	2.30E-04				
SOD1	FBS	2.84749	2	1.424	5339	< .001	0.427	0.998
	Oxygen	1.9608	2	0.98	3676	< .001	0.294	0.998
	FBS ✻ Oxygen	1.86311	4	0.466	1747	< .001	0.279	0.997
	Residuals	0.0048	18	2.67E-04				
SOD2	FBS	11.53479	2	5.767	20763	< .001	0.742	1
	Oxygen	0.86214	2	0.431	1552	< .001	0.055	0.994
	FBS ✻ Oxygen	3.15164	4	0.788	2836	< .001	0.203	0.998
	Residuals	0.005	18	2.78E-04				
SOD3	FBS	45.3579	2	22.68	92778	< .001	0.876	1
	Oxygen	2.35512	2	1.18	4817	< .001	0.045	0.998
	FBS ✻ Oxygen	4.04441	4	1.01	4136	< .001	0.078	0.999
	Residuals	0.0044	18	2.44E-04				

* $p < 0.05$

Supplementary Table S7. Interaction effect of FBS and oxygen concentration on the regulation of myogenic regulators by SMCs differentiation medium.

Genes		Sum of	df	Mean Square	F	p	η²	η²p
**F10 DM**	FBS	0.42503	2	0.213	390	< .001	0.201	0.977
MYF5	Oxygen	1.09081	2	0.545	1002	< .001	0.516	0.991
	FBS ✻ Oxygen	0.5865	4	0.147	269	< .001	0.278	0.984
	Residuals	0.0098	18	5.44E-04				
**F10 DM**	FBS	1.40643	2	0.7032	5933	< .001	0.725	0.998
MYOD1	Oxygen	0.09283	2	0.0464	392	< .001	0.048	0.978
	FBS ✻ Oxygen	0.43906	4	0.1098	926	< .001	0.226	0.995
	Residuals	0.00213	18	1.19E-04				
**F10 DM**	FBS	1.6091	2	0.8045	940.4	< .001	0.552	0.991
MYOG	Oxygen	1.0007	2	0.5003	584.8	< .001	0.343	0.985
	FBS ✻ Oxygen	0.2902	4	0.0726	84.8	< .001	0.1	0.95
	Residuals	0.0154	18	8.56E-04				
**F10 DM**	FBS	3.2132	2	1.6066	2507	< .001	0.694	0.996
MYF6	Oxygen	1.0805	2	0.5402	843	< .001	0.233	0.989
	FBS ✻ Oxygen	0.3225	4	0.0806	126	< .001	0.07	0.965
	Residuals	0.0115	18	6.41E-04				
**F10 DM**	FBS	1.6469	2	0.82345	453	< .001	0.47	0.981
MYH1	Oxygen	0.8412	2	0.4206	231	< .001	0.24	0.963
	FBS ✻ Oxygen	0.9816	4	0.2454	135	< .001	0.28	0.968
	Residuals	0.0327	18	0.00182				
**F10 DM**	FBS	2.1531	2	1.07656	830	< .001	0.66	0.989
MSTN	Oxygen	0.4874	2	0.24369	188	< .001	0.149	0.954
	FBS ✻ Oxygen	0.5987	4	0.14968	115	< .001	0.184	0.962
	Residuals	0.0233	18	0.0013				
**DMEM/F12 DM**	FBS	0.1624	2	0.0812	76.9	< .001	0.004	0.895
MYF5	Oxygen	34.8731	2	17.43656	16518.8	< .001	0.932	0.999
	FBS ✻ Oxygen	2.3438	4	0.58594	555.1	< .001	0.063	0.992
	Residuals	0.019	18	0.00106				
**DMEM/F12 DM**	FBS	3.4503	2	1.725	2948	< .001	0.649	0.997
MYOD1	Oxygen	1.0611	2	0.531	907	< .001	0.2	0.99
	FBS ✻ Oxygen	0.7956	4	0.199	340	< .001	0.15	0.987
	Residuals	0.0105	18	5.85E-04				
**DMEM/F12 DM**	FBS	2.8835	2	1.442	1582	< .001	0.355	0.994
MYOG	Oxygen	4.58	2	2.29	2513	< .001	0.565	0.996
	FBS ✻ Oxygen	0.6313	4	0.158	173	< .001	0.078	0.975
	Residuals	0.0164	18	9.11E-04				
**DMEM/F12 DM**	FBS	1.4	2	0.7	566	< .001	0.162	0.984
MYF6	Oxygen	1.8853	2	0.94267	762	< .001	0.218	0.988
	FBS ✻ Oxygen	5.3219	4	1.33047	1076	< .001	0.617	0.996
	Residuals	0.0223	18	0.00124				
**DMEM/F12 DM**	FBS	1.4356	2	0.718	941	< .001	0.042	0.991
MYH1	Oxygen	31.7912	2	15.896	20834	< .001	0.938	1
	FBS ✻ Oxygen	0.667	4	0.167	219	< .001	0.02	0.98
	Residuals	0.0137	18	7.63E-04				
**DMEM/F12 DM**	FBS	4.992	2	2.496	950.52	< .001	0.575	0.991
MSTN	Oxygen	3.595	2	1.79751	684.53	< .001	0.414	0.987
	FBS ✻ Oxygen	0.0524	4	0.01311	4.99	0.007	0.006	0.526
	Residuals	0.0473	18	0.00263				
