# Supplementary figures and images for: Effect of Serum and Oxygen on the In Vitro Culture of Hanwoo Korean Native Cattle-Derived Skeletal Myogenic Cells Used in Cellular Agriculture
Source: Foods. 2023 Mar 24;12(7):1384. doi: 10.3390/foods12071384 (PMC10093918; doi:10.3390/foods12071384)

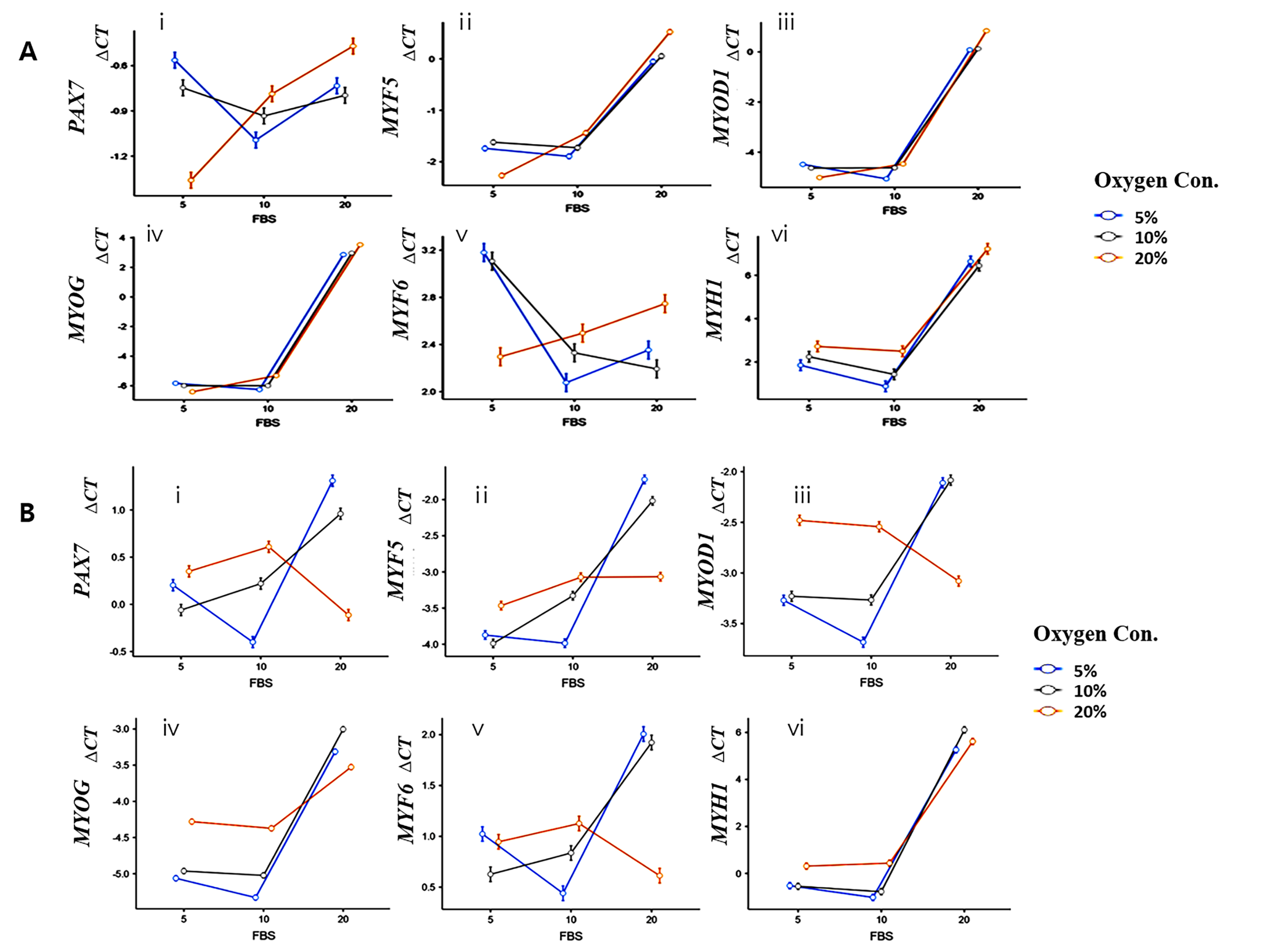

Supplement: Supplementary file 1 [file foods-12-01384-s001.zip › Supple fig S1.tif]

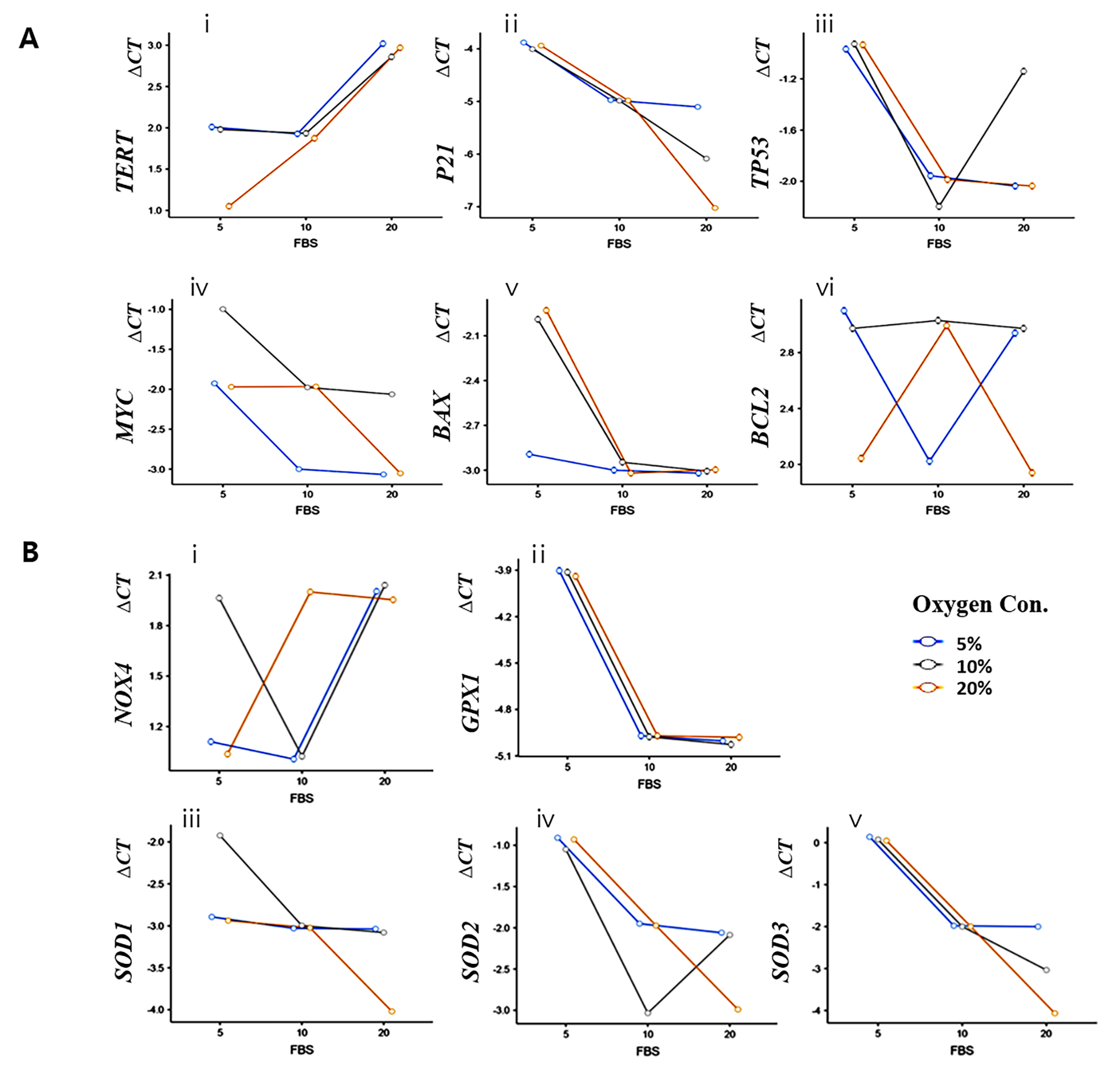

Supplement: Supplementary file 1 [file foods-12-01384-s001.zip › Supple fig S2.tif]

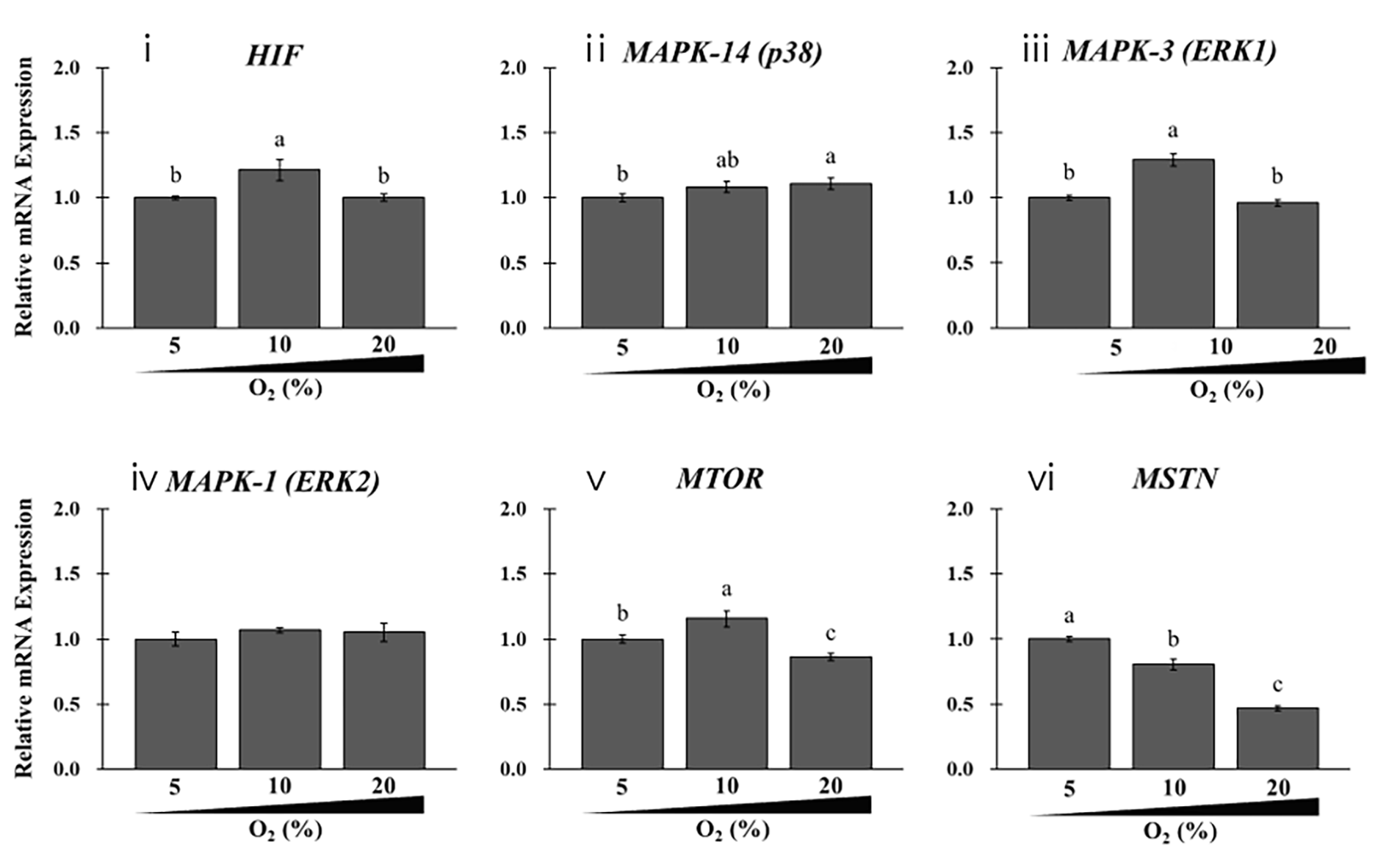

Supplement: Supplementary file 1 [file foods-12-01384-s001.zip › Supple fig S3.tif]

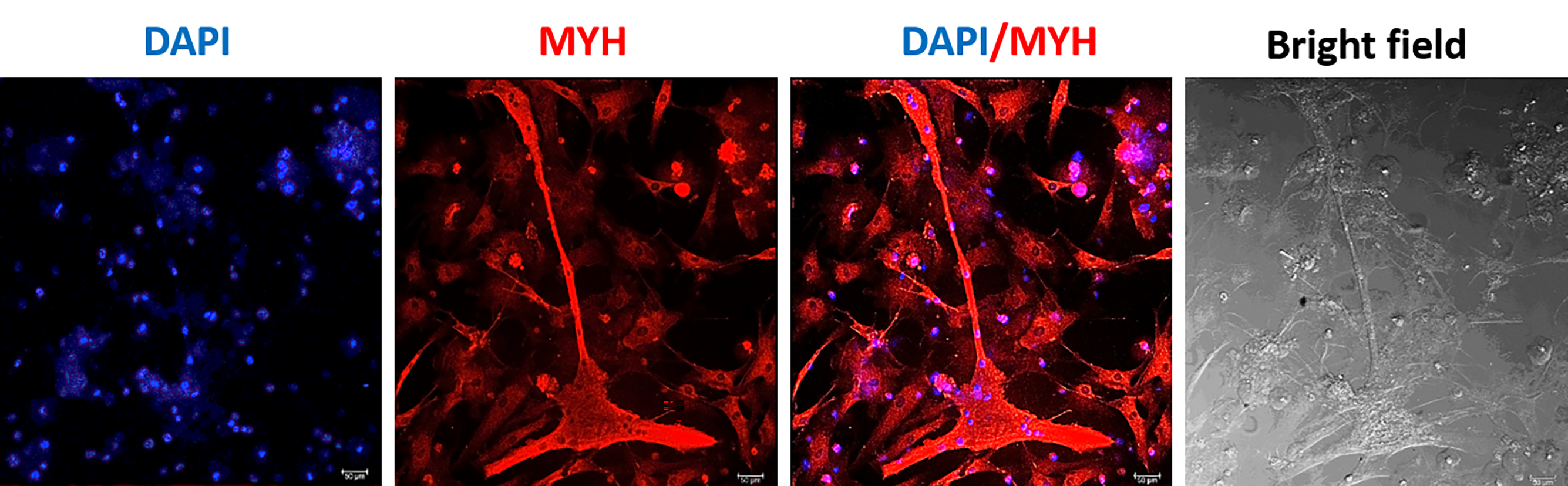

Supplement: Supplementary file 1 [file foods-12-01384-s001.zip › Supple fig S4.tif]
